# Dimensions

**DOI:** 10.5195/jmla.2019.695

**Published:** 2019-07-01

**Authors:** Roxann W. Mouratidis

**Affiliations:** Scholarly Communications Librarian, Charlotte Edwards Maguire Medical Library, College of Medicine, Florida State University, Tallahassee, FL, roxann.mouratidis@med.fsu.edu

## PURPOSE

Dimensions is a linked-research data platform that aims to reveal connections between research and its scholarly outputs. By partnering with several publishers, vendors, and organizations, Dimensions combines a robust citation database with a research analytics suite to track the impact of research across its life cycle. Dimensions allows one to locate specific papers and find related papers, researchers, and scholarly products in a particular field of research.

## CONTENT

The Dimensions scholarly database includes research articles, books, chapters, awarded grants, patents, clinical trials, and policy documents. The metadata for this database is harvested from sources such as CrossRef, PubMed, Directory of Open Access Journals, Open Citation Data, clinical trial registries, patent offices, and over 100 publishers [1, 2]. In some cases, full-text data are indexed from open sources such as PubMed Central and arXiv, providing greater discovery and access capabilities than other citation databases [3].

## INTENDED AUDIENCE

Dimensions comprises an underlying citation database and research analytics suite; therefore, the intended audience is broad and is likely to appeal to librarians, researchers and academics, university administrators, research officers, and funders. Using the Dimensions platform, it is possible to (1) quickly see connections between scholarly objects, researchers, funders, and institutions; (2) retrieve specific article-level metrics (e.g., citation counts, alternative metrics); and (3) discover which fields of research are active and growing. These features would be attractive to a wide group of stakeholders who are interested in tracking the impact of research output.

## FEATURES

Dimensions integrates established metrics sources to demonstrate the impact, attention, and influence of a particular body of research among the academic and wider community. Traditional metrics such as citation counts are available and are calculated from the reference list in all publications indexed by Dimensions. Additionally, Dimensions includes the Field Citation Ratio and the Relative Citation Ratio, which attempt to determine the relative citation performance of an article compared to similar articles in the same field.

Instead of promoting journal-level impact measures such as the journal impact factor, Dimensions incorporates article-level measures to demonstrate the influence of an article on its own terms, independent of where it was published. Dimensions complements traditional metrics by partnering with Altmetric to leverage their alternative metrics or “altmetrics” data to reveal the amount of attention an individual article has received. With altmetrics, it is possible to determine how many times an article was shared on a social media platform, news outlet, blog, or website.

## USABILITY

### Publications database

Learning how to navigate the Dimensions publication database requires minimal time and effort. The search techniques are not designed to be complex, so one can type in a topic, title, or author name into the main search box and quickly retrieve relevant information. One has the option to perform different types of searches; for example, one can search full-text data, titles, and abstracts or by digital object identifier (DOI).

Even though one is not required to create a user account with Dimensions to search the publications database, registration is required if one would like to save one’s searches as “Favorites” or export search results. Search results can also be sorted by relevance, publication date, citations, or altmetric score.

Search results can be further refined through the use of filters. One can narrow results by publication year, fields of research, or publication type. Additionally, filters can be applied to limit the search to a specific researcher, publications directory source, journal title, or type of open access content.

### Analytics suite

Altmetric scores and citation counts are listed in the publication record in Dimensions. From the publication record, one can click on the altmetric score to see specific details such as how many times and where the article was shared. Also, any citations of the article are listed in the publication record along with its references and any related grants.

Dimensions also has a menu to display its “Analytical Views.” In this view, it is possible to quickly view related fields of research, journal titles, or researchers. The “Overview” tab provides a graphic visualization of publication trends over time related to a specific search. Through further exploration, one can determine the number of publications and citations retrieved by one’s search, as well as the percentage of cited or not cited items, and the number of citations per publication.

## COMPARISON WITH OTHER DATABASES

Dimensions encompasses similar features that are available in other scholarly databases, although in this reviewer’s opinion, no single database is directly comparable. Dimensions is similar to Web of Science, Scopus, and Google Scholar in terms of indexing a wide range of content types across many research fields beyond biomedical sciences. As with these databases, Dimensions provides citation metrics but expands these metrics to include altmetrics.

Dimensions lacks the precise advance searching of PubMed. However, it is easier with Dimensions to see bigger picture activity occurring across research areas, institutions, and funders, and among researchers.

Another feature that sets Dimensions apart from other databases is its ability to seamlessly connect one to full-text portable document format (PDF) articles. Through a partnership with ReadCube Papers, it is possible to access, read, and save journal articles without having to leave the Dimensions platform. This type of seamless access parallels that of SciHub, where individuals can quickly access full-text content through illicit practices. However, unlike SciHub, Dimensions content is provided via legal open access sources and institutional subscriptions.

## PRICING LEVELS

Dimensions has been described as utilizing a “freemium” business model [3]. The publication database component of Dimensions is free, and registration is not required to search for articles and authors or to discover links to grants and patents. Nevertheless, registration is encouraged in the event that one’s institution enables a paid feature such as seamless access to institutionally licensed content.

**Figure 1:**
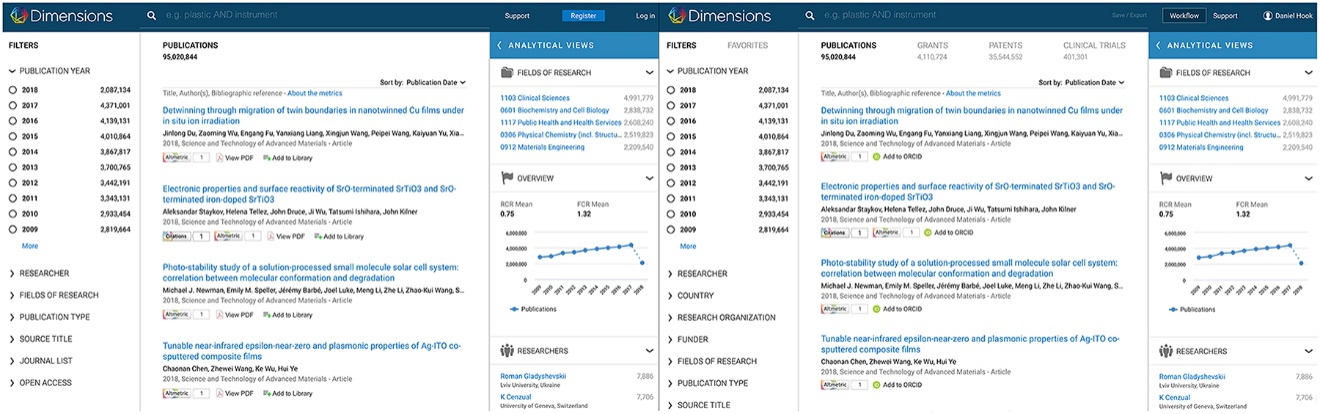
Free (left) and paid (right) versions of Dimensions side-by-side Source: Hook DW, Porter SJ, Herzog C [1], distributed under a CC-BY license.

Access to the research analytics component of Dimensions is limited to subscribing institutions. Called Dimensions Plus, this paid service allows universities and institutions to search for non-publication content such as grants, clinical trials, and patents. Dimensions Plus also has additional analytical capabilities and allows one to conduct visualizations and comparisons that are not included in the free version (Figure 1).

## CONCLUSION

Dimensions aims to put research in context using an underlying linked-data structure. By connecting research outputs, Dimensions allows one to find related publications, researchers, grants, and more. Through the integration of multiple publication metadata sources, article impact metrics, and in some cases, seamless access to full-text, Dimensions is a unique tool that benefits librarians, researchers, funders, and administrators in their work and research analysis.
